# Why Is Psychiatry so Intimately Linked to the Brain?

**DOI:** 10.3389/fpsyt.2022.872957

**Published:** 2022-06-02

**Authors:** Christophe Gauld, Pierre Fourneret, Jean-Arthur Micoulaud-Franchi

**Affiliations:** ^1^Service de Psychopathologie du Développement de l'Enfant et de l'Adolescent, Hospices Civils de Lyon & Université de Lyon 1, Lyon, France; ^2^UMR CNRS 8590 IHPST, Sorbonne University, Paris, France; ^3^University Sleep Clinic, Service of Functional Exploration of the Nervous System, University Hospital of Bordeaux, Bordeaux, France; ^4^USR CNRS 3413 SANPSY, University Hospital Pellegrin, University of Bordeaux, Bordeaux, France

**Keywords:** neurosciences, network, sociology, path dependence, brain sciences, challenges, future

**Graphical Abstract:**
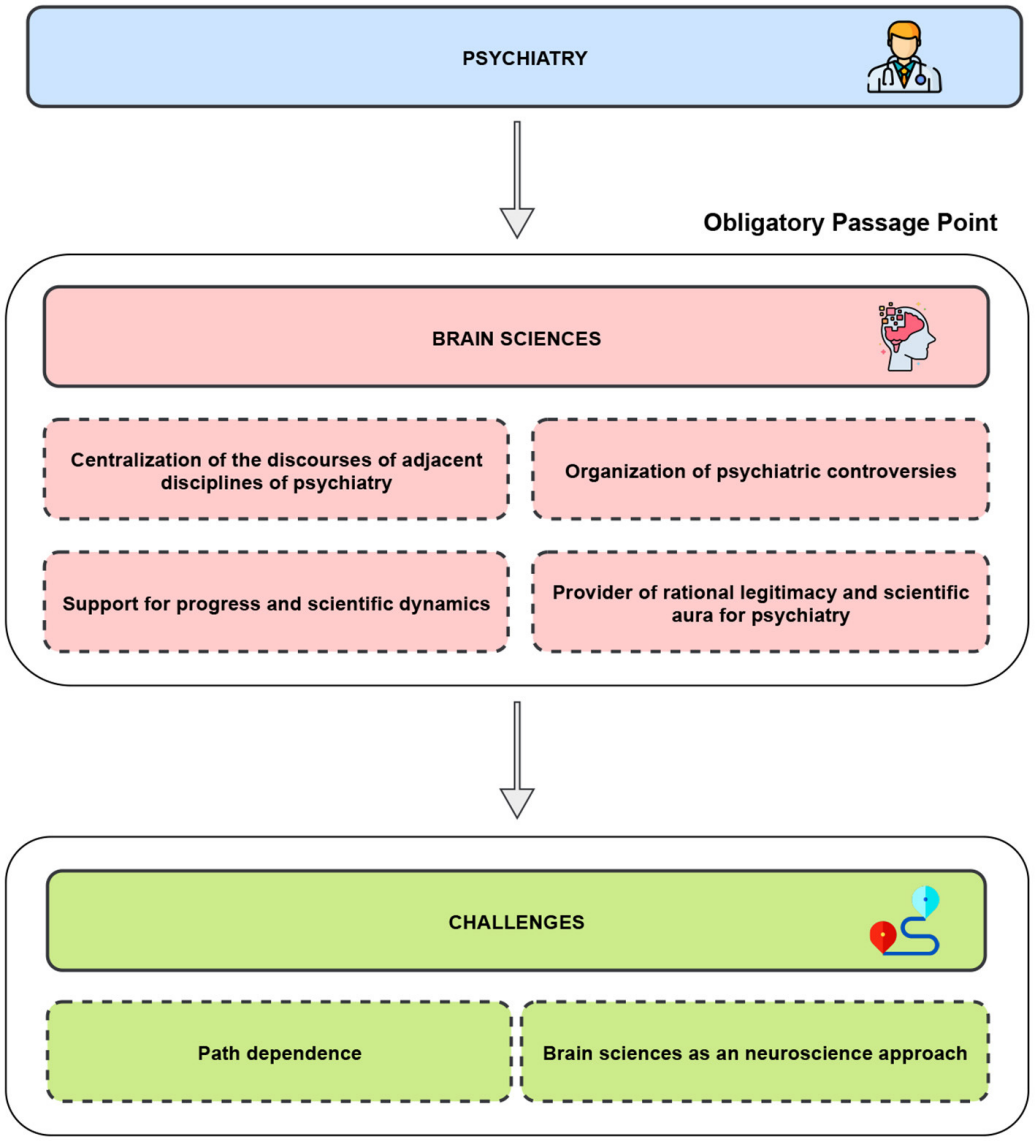


## Introduction

In psychiatry, clinical heterogeneity of mental disorders is the rule rather than the exception. As an illustration, 1,030 symptom profiles have been isolated for depression, of which 501 appear only once (1). In addition to such wide interindividual variability, with more than 50% of patients usually given least two diagnoses, one can also observe strong comorbidity (2, 3). This heterogeneity of psychiatry presents many difficulties, both in terms of reliability, construct validity of the disorders, or in terms of the nature and type of measures used in psychometry to characterize psychiatric disorders (4).

One of the main responses to these challenges has been reliance on an element that is apparently central to psychiatry: the reference to the brain, or “brain sciences.” Until now, the pivotal position of the brain in psychiatry can be explained by the descriptive (and sometimes explanatory) force of its concepts and neurocognitive models applied to psychopathology. Indeed, references to the brain have allowed many advances in psychopharmacology, cognitive remediation or in the ability to predict particular types of physiopathological models at the origin of psychiatric disorders (e.g., predictive Bayesian computational simulations). The pivotal position of brain sciences can also be explained by a set of historical, conceptual, normative, empirical or technological factors, e.g., progress in statistical methods, importance of experimentation or the desire to propose translational evidence between biology and clinical practice.

Brain sciences do therefore not only provide the latest scientific evidence to the discipline of psychiatry, but they represent a fundamental basis upon which the psychiatric field relies. In other words, psychiatry cannot separate itself from brain sciences, even if its conclusions proved to be questionable. Consequently, psychiatry and brain sciences are closely linked by a multitude of sociological, organizational, societal and systemic factors.

This importance of brain sciences for psychiatry is particularly consistent with the actor-network theory (5). This sociological theory defines an Obligatory Passage Point (OPP) as an object or a situation which brings together and aggregates the actors within a social network (defined by a set of problematizations organized around this object) (5). Therefore, brain sciences constitute an OPP structuring and harmonize a large number of current heterogeneous fields of psychiatry. They offer a sociological stabilization of the discipline and constitute *de facto* a consensual object anchored within the constantly evolving field of psychiatry. However, note that this OPP could only be considered when we consider all psychiatry as a whole. As soon as only one of the sub-specialties of psychiatry (e.g., psychoanalysis or Child Psychiatry) is considered, the influence of this OPP is minimized.

## An Obligatory Passage Point for Psychiatry

The notion of an OPP could give rise to at least four reasons for which such a reference to the brain still exerts a strong influence on psychiatry.

First, brain sciences allow psychiatry to centralize the discourses of its adjacent disciplines, as diverse and distant from each other as genetics, molecular biology, biochemistry, medical imaging or applied statistics. This centralization of disciplines that are adjacent to psychiatry allows their didactic integration into clinical practice. The relevance of brain sciences for psychiatry is provided by the capacity to study a single organ, which is functionally described at different levels (6).

Secondly, brain sciences could help to elucidate psychiatric controversies. By providing scientific models, brain sciences centralize the struggle between the clinicians and the scattered data they accumulate. They provide insight into which arguments could be most suitable to inform a scientific or clinical question. Indeed, the brain's centrality for these controversies offers a robust methodological foundation for psychiatry and provides a reference point (and even, a benchmark) to the field (7).

Thirdly, brain sciences are considered as a set of tools for progress and scientific dynamics, thereby strengthening the integration of psychiatry into the field of evidence-based medicine. In other words, brain science can answer many of the clinical and therapeutic decisions.

Fourthly, because of the guarantee of veracity engendered by the important credibility of brain sciences (related to its plausibility, reliability, scientific and historical influences), brain sciences finally confer a rational legitimacy and a scientific aura on psychiatry, which may have suffered in the past from its clinical interpretations based on the subjectivity of patients (5).

Thus, the fact that psychiatry refers so much to the brain allows for an organization of knowledge production techniques, as well as for a centralization of communication channels and debates, which in turn, support research activity and the definition of standards for clinical practice.

## Discussion

However, identifying the brain sciences as an OPP for psychiatry presents at least two challenges: (1) not to suffer a path dependence; (2) and to keep in mind that the OPP of psychiatry has evolved and is much larger than solely brain sciences are today.

Thus, first, brain sciences could lead to a “path dependence” for psychiatry. Path dependence is a conceptual lockdown that steers the discipline in a particular direction, potentially hampering the development of parallel pathways (e.g., the search for other perspectives potentially more relevant to psychiatry than neuroscience) (8). Such notion of path dependence partly explains the influx of funding for research (e.g., psychopharmacology), while other research avenues such as epidemiological or psychotherapeutic investigations have been significantly underfunded.

Secondly, this OPP centered around brain sciences could evolve in coming years. It seems necessary to consider this development. Even if the current centrality to the brain in psychiatry is far from disappearing, the direct reference of the brain is currently losing its central leadership position. Specifically, the field of contemporary neuroscience is much larger than the field of brain sciences (6). The former constitutes a highly heterogeneous field, which seeks to answer the complex questions research within psychiatry poses. For instance, questions aim to explore psychiatric profiles through the notion of *equifinality* (referring to the question of how different processes can lead to the same diagnosis) and *multifinality* (asking how similar processes can lead to different diagnoses) (9). Thus, they complete and enrich the collection of diagnostic, predictive and prognostic biomarkers both qualitatively (e.g., with markers of various nature such as exposomics and cultural markers) and quantitatively (e.g., with ecological momentary big data) (10). Contemporary neuroscientific developments also lead to the development of precision psychiatry, defined as “the adaptation of medical treatment to the individual characteristics of each patient.” Precision psychiatry offers computational and dynamic models, allowing to overcome or resolve the reproducibility pitfall classically opposed to the validity of psychiatric disorders, as well as for refinement and emergence of new nosologies or stratification based on various biomarkers. These emerging proposals from contemporary neurosciences attempt to break free from the direct study of the different scales of the brain, by building increasingly complex perspectives, for instance, using mathematical language as an alternative to traditional narrative models of psychiatry, contextual, environmental or cultural elements.

In conclusion, it is obvious that the field of psychiatry is reliant upon knowledge of the brain, but this extended brain must be the image of contemporary neuroscience, i.e., it needs to be construed as heterogeneous, multiple, flexible and dynamic. In this way, other disciplines and theories could also influence debates on psychiatry as does the OPP constituted by the brain sciences: it is undoubtedly the set of disciplines assembled around this OPP which constitutes the explanatory core of the evolution of psychiatry until this day and throughout the future.

## Author Contributions

CG: writing, original draft preparation, conceptualization, methodology, and editing. PF: supervision, reviewing, and validation. J-AM-F: conceptualization, writing, supervision, methodology, reviewing, and validation. All authors contributed to the article and approved the submitted version.

## Conflict of Interest

The authors declare that the research was conducted in the absence of any commercial or financial relationships that could be construed as a potential conflict of interest.

## Publisher's Note

All claims expressed in this article are solely those of the authors and do not necessarily represent those of their affiliated organizations, or those of the publisher, the editors and the reviewers. Any product that may be evaluated in this article, or claim that may be made by its manufacturer, is not guaranteed or endorsed by the publisher.

## Abbreviations

OPP: obligatory passage point.
